# The parotid gland, an unusual site of colorectal cancer metastasis

**DOI:** 10.3332/ecancer.2023.1560

**Published:** 2023-06-15

**Authors:** Paula Isabel G Francoa, Anna Pascual-Panganiban

**Affiliations:** Section of Medical Oncology, St Luke’s Medical Center, 1112 Quezon City, Philippines; a https://orcid.org/0000-0002-4178-7722

**Keywords:** metastasis, colorectal cancer, parotid gland, case report

## Abstract

Colorectal cancer commonly metastasises to the liver, peritoneum and lungs. With disseminated disease, it can spread to more unusual sites. Parotid gland metastasis usually originates from head and neck malignancies. We present a case of stage IV sigmoid colon adenocarcinoma with metastases to the left parotid. The patient was a 53-year-old Filipino man diagnosed with stage IV sigmoid adenocarcinoma with liver metastases in June 2021. He underwent laparoscopic sigmoidectomy and received eight cycles of chemotherapy with capecitabine and oxaliplatin with partial response of his liver lesions. Capecitabine monotherapy was then maintained. On September 2022, he experienced persistent left facial pain, with no relief after dental tooth extraction and antibiotics. A computed tomography (CT) scan revealed a 5 × 7 × 6 cm inhomogenous mass in the left parotid with destruction of the mandible. A fine needle biopsy was consistent with a high-grade carcinoma. After multidisciplinary discussions, a repeat core needle biopsy was deemed necessary to proceed with immunohistochemistry. With strong positivity for cytokeratin 20 (CK20), carcinoembryonic antigen, special AT-rich sequence-binding protein 2 and CAM 5.2, and weak positivity for CK7, the parotid mass was diagnosed as metastatic adenocarcinoma from the colon. He then received palliative radiation to the parotid mass for pain control. A gastrostomy tube was also inserted for nutritional support. Treatment with next-line chemotherapy (FOLFIRI regimen) was planned. Unfortunately, he contracted COVID-19 pneumonia and succumbed to respiratory failure. Pursuing the histologic diagnosis of this uncommon area of metastasis was necessary for appropriate treatment planning. Fostering multidisciplinary collaboration throughout the complex aspects of cancer care requires patient advocacy, leadership and effective communication. For our patient, it was essential to coordinate with surgery and pathology to maximise the diagnostic yield of a repeat biopsy while minimising complications and treatment delays.

## Introduction

Colorectal cancer metastasises through lymphatic and haematogenous means, primarily to the liver, peritoneum or lungs [1]. The average 3-year overall survival rate for metastatic colorectal cancer approaches 20%, with extrahepatic disease portending a dismal prognosis [2]. Involvement of rare sites of metastases such as the skeletal muscle, heart and thyroid occurs with uncontrolled, widespread disease [3]. Metastases to the parotid gland, in itself, is rare with an incidence of 8.1% in a series of 6,000 patients [4]. With a higher incidence of primary sites being from the head and neck region, metastatic spread of colorectal cancer to the parotid gland is extremely rare [4, 5]. As of writing this report, only five other known cases have been documented [1, 3–5]. Moreover, metastatic lesions in the parotid gland can occur in the paraglandular or intraglandular space, making it difficult to distinguish from the primary tumours of the gland itself [5]. Judicious biopsies, immunostaining and pathologic review in the context of clinical information facilitate the timely diagnosis of rare sites of metastases and its subsequent treatment.

We present a rare case of colorectal cancer metastases to the parotid. Herein, we also highlight the need for ensuring a good diagnostic yield when repeating biopsies and for concerted multidisciplinary efforts in the management of uncommon presentations of metastatic colorectal cancer.

## Case presentation

### History

The patient is a 53-year-old Filipino man diagnosed with sigmoid adenocarcinoma with *de novo* liver metastasis in June 2021. He initially underwent laparoscopic sigmoidectomy to relieve his partial gut obstruction. Although it is not the recommended first-line treatment for metastatic colon cancer, the patient was then treated with capecitabine at 1,250 mg/m^2^ twice a day for 14 days and oxaliplatin at 130 mg/m^2^ at 21-day cycles. Due to financial constraints and lack of local reimbursement, molecular testing for kirsten rat sarcoma viral oncogene homolog, neuroblastoma RAS viral oncogene homolog, v-raf murine sarcoma viral oncogene homolog B1 mutations and mismatch repair deficiency or microsatellite instability was not pursued. Furthermore, additional targeted treatments such as Cetuximab or Bevacizumab or immunotherapy are an out-of-pocket expense locally. After eight cycles of capecitabine and oxaliplatin, a partial response by response evaluation criteria in solid tumors criteria of his liver lesions was achieved despite a minimal increase in his carcinoembryonic antigen (CEA) (42 ng/mL) from baseline (33 ng/mL). He was then maintained on capecitabine monotherapy. On September 2022, he experienced persistent left facial pain, with no relief after dental tooth extraction and 2 weeks of oral antibiotics.

### Work-up and diagnostics

**Figure 1.**
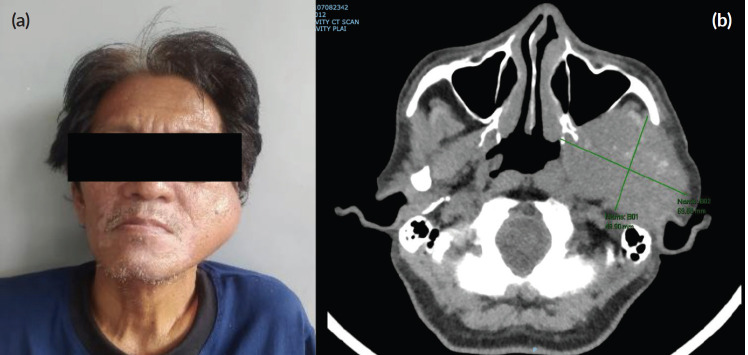
(a): Rapidly growing left facial mass associated with occasional pain and trismus. (b): CT scan of the left parotid mass with osseous destruction of the mandible.

Despite the patient’s rapidly growing left facial mass (Figure 1a), consultation with his medical oncologist was delayed due to inadequate health-seeking behaviour and financial difficulties. A neck and oral cavity contrast-enhanced CT scan revealed a 5 × 7 × 6 cm inhomogenous mass in the left parotid with osseous destruction of the mandible (Figure 1b).

**Figure 2.**
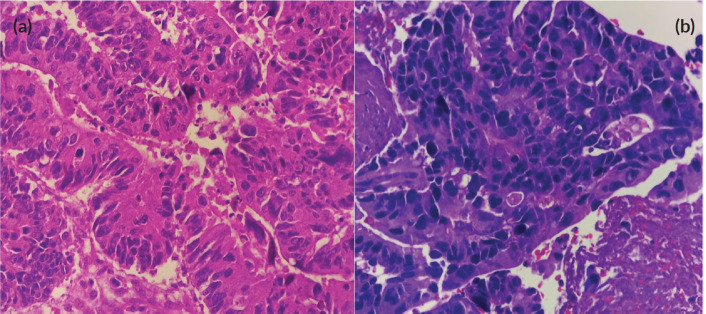
(a): Primary sigmoid adenocarcinoma (haematoxylin and eosin stain, at 40× magnification). (b): High-grade carcinoma of the parotid gland (haematoxylin and eosin stain, at 40× magnification). Both images show disorganised glandular patterns with high-grade nuclear features.

Fine needle biopsy of the mass showed papillary, solid and disorganised glandular patterns with high-grade nuclear features with pleomorphic nuclei – consistent with a high-grade carcinoma, and not purely adenocarcinoma (Figure 2a and b). A repeat chest and abdominal contrast-enhanced scan also revealed progression of his liver lesions (multiple enhancing inhomogenous masses with the largest progressing in size, now measuring 7 × 5 cm from 4 × 5 cm previously). Differential diagnoses of the new parotid malignancy included metastatic adenocarcinoma from the colon as well as a new head and neck primary malignancy, particularly, a mucoepidermoid carcinoma of the parotid gland. Despite concerns regarding poor prognosis, the patient and his team of doctors agreed that a correct diagnosis and proper palliative treatment must be pursued. After multidisciplinary meetings with surgery, radiation oncology and pathology, a repeat biopsy through a core needle was deemed necessary to proceed with immunohistochemistry stains. With strong positivity for cytokeratin 20 (CK20), special AT-rich sequence-binding protein 2 (SATB2) and CAM 5.2, and weak positivity for CK7, the parotid mass was diagnosed as a metastatic adenocarcinoma from the colon.

### Outcome/treatment

He then received palliative local radiation at 20 Gy given in five fractions for pain control. A gastrostomy tube was also inserted for nutritional support. With a fair performance status at a Karnofsky grade of 70, treatment with FOLFIRI (combination chemotherapy with 5-fluorouracil, leucovorin, and irinotecan) was then planned. However, the patient contracted severe COVID-19 pneumonia before starting treatment and eventually died of respiratory failure after 3 weeks of hospital admission.

## Discussion

Approximately one-third of patients diagnosed with *de novo* metastatic colorectal cancer present with synchronous liver metastasis [2]. Apart from the peritoneum and the lungs, other rare sites of metastases (skeletal muscle, heart, cutaneous, spleen, adrenal gland and parotid) have been documented [1, 3–5]. In some of these cases, metastasectomy and subsequent systemic treatment were successfully instituted. However, the majority of the reported cases presented with concurrent or imminent widespread metastases, portending a poor prognosis [3]. One patient with metastasis to the parotid already had poor performance status upon diagnosis, and thus received palliative radiation for pain control and capecitabine-irinotecan until disease progression [4]. Another underwent a successful surgical resection (right total parotidectomy and neck dissection) and subsequent treatment with five cycles of capecitabine-oxaliplatin [5]. In both cases, clinical deterioration within 6 months prompted the patients to eventually undertake the best supportive care [4, 5].

Approximately 20% of parotid gland tumours are of malignant origin [6] and only about a quarter of these are metastatic, originating from malignancies of the head and neck, and of squamous carcinoma or melanoma histology. Tumours originating from below the clavicle are even more rare [5]. Apart from a metastatic disease from the colon, the other major differential of the health care team was a primary high-grade mucoepidermoid carcinoma of the parotid. Patients already diagnosed with cancer can develop another *de novo* malignancy depending on inherited, environmental and even iatrogenic risk factors (treatment modalities such as chemotherapy and radiation). Moreover, the atypical location alerted us to the possibility of a second primary cancer [7]. Mucoepidermoid carcinoma constitutes 35% of all salivary malignancies and frequently occurs in the parotid. A higher grade is predictive of aggressive and infiltrative behaviour as well as poor prognosis. Histologically, these are characterised by irregular tumour patterns (papillary, solid and glandular growth patterns) that invade parenchyma and demonstrate separate tumour islands [8].

Our patient’s initial biopsy was done through fine needle aspiration. The smears were able to demonstrate highly cellular aspirates, high-grade nuclear features with pleomorphic nuclei and prominent nucleoli. These findings were consistent with a high-grade carcinoma, but it did not rule out an adenocarcinoma of metastatic origin [8]. In lower-income laboratories, core tissue specimens or cell blocks are needed to be able to produce completed immunohistochemical stains on paraffin-embedded tissue. Due to the paucity of material, immunohistochemistry is rarely performed on tissue stains obtained through fine needle aspiration. In fact, an average of four immunohistochemical markers are done on tissue obtained through core needle biopsies as compared to an average of only one marker with fine needle aspiration biopsies (FNAB) [9]. We know that an FNAB is the preferred diagnostic procedure when malignancy of a neck mass or cervical lymph node is suspected. However, in situations where an FNAB is non-diagnostic, a core biopsy must be obtained rather than an open biopsy which is recommended only if definitive surgical management is planned [10]. Establishing timely tissue diagnosis remains the gold standard especially when considering a possible secondary malignancy [7].

Immunostaining is invaluable in the accurate diagnosis of primary tumours and their rare sites of metastases. Typically, the immunoprofile of colon adenocarcinomas is CK 20 positive, CEA-positive and CK 7-negative (Table 1). Expression of CK 7 is seen in ductal and glandular epithelia, such as the salivary glands, pancreas, ovary and endometrium. However, a minor subset of colorectal carcinomas may also be focally or weakly CK 7 positive, as seen in our patient. Increased CK 7 expression is indicative of a poorer prognosis in colorectal cancer patients [11]. Our patient’s parotid tissue was also positive for the transcription factor SATB2 and CKCAM 5.2, whose presence are often restricted to the lower gastrointestinal tract. These increase the predictive value (from 93% to almost 99%) of CK20-positive/CK7-negatively expressing tissues as originating from the colon [12].

The treatment plan and prognosis for our patient would have been markedly different if the parotid mass had been diagnosed as a high-grade, unresectable primary parotid carcinoma. Salivary gland carcinomas respond poorly to standard chemotherapy. Without complete surgical resection as an option, definitive radiotherapy or systemic therapy with radiation would have been offered to our patient. Testing for other biomarkers such as androgen receptor, Her2 and neurotrophic tyrosine receptor kinase gene for targeted treatment would also have been considered [10]. Novel agents against these biomarkers and definitive radiation would have been offered together with palliative FOLFIRI chemotherapy if our patient did indeed have two primary malignancies (an unresectable parotid gland carcinoma and a metastatic colorectal cancer).

Finally, a salient challenge in the management of our patient was the need for coordinated cancer care. The work-up of his parotid gland mass and instituting other supportive treatments (insertion of a gastrostomy tube for nutrition and initiating palliative radiotherapy for pain control) required collaboration with physicians from different subspecialties. Without effective coordination, patients experience treatment delays, worse quality of life and poorer outcomes [13, 14]. According to a meta-analysis, formal and informal mechanisms of physician collaboration improve cancer outcomes in terms of transitioning from neoadjuvant treatment to timely surgery, and adjuvant therapy [13]. Apart from collaboration among specialists, the bigger part of holistic care for patients, in general, is advocating for their goals and preferences [14]. During our multidisciplinary meetings, the patient and his family were apprised of his prognosis. The option of best supportive care was also presented. However, apart from facial pain making food consumption difficult, the patient had a fair performance status. The patient verbalised their intent to pursue proper diagnosis and treatment of his parotid mass. The healthcare team then made a concerted effort to respect the patient’s informed decision.

## Conclusion

Differentials for aggressive parotid gland lesions include primary salivary gland malignancy (such as mucoepidermoid carcinoma) or metastases from a head and neck primary cancer. Parotid gland metastasis from a colorectal primary malignancy is possible, especially with widespread disease. Subsequently, a high index of suspicion is needed to recognise unusual sites of metastases earlier. Pursuing the accurate diagnosis of this uncommon area of metastasis was necessary for proper treatment planning. This involves coordinating between pathology and surgery to establish the most appropriate means of obtaining tissue that has the greatest diagnostic yield while minimising complications and treatment delays. Finally, fostering multidisciplinary collaboration throughout the complex aspects of cancer care requires patient advocacy, leadership and effective communication.

## Statement of informed consent

Informed consent was obtained from the patient’s family regarding the writing and publication of this article.

## Conflicts of interest

The authors declare that they have no competing interests.

## Funding

No funding was received for this research.

## Figures and Tables

**Table 1.** Colon and parotid adenocarcinomas and their associated immunostaining results [12].

Primary site	CK 7	CK 20	CEA	SAT B2	CAM 5.2
Colon	Negative/positive (minority)	Positive	Positive	Positive	Positive
Parotid	Positive	Negative	Negative	Negative	Negative

CEA: carcinoembryonic antigen, CK: cytokeratin, SATB2: special AT-rich sequence-binding protein 2
